# *Tetracarpidium conophorum* ameliorates oxidative reproductive toxicity induced by ethanol in male rats

**DOI:** 10.1186/s12906-015-0960-5

**Published:** 2015-12-18

**Authors:** S. F. Akomolafe, G. Oboh, A. A. Akindahunsi, A. J. Afolayan

**Affiliations:** Department of Biochemistry, Ekiti State University, P.M.B 5363, Ado, Ekiti Nigeria; Department of Biochemistry, Federal University of Technology, P.M.B 704 Akure, Nigeria; Department of Botany, University of Fort Hare, X1314 Alice, South Africa

**Keywords:** MDA, Antioxidant enzymes, Reproductive tissues, Infertility, Medicinal plant

## Abstract

**Background:**

*Tetracarpidium conophorum* (Mull. Arg.) Hutch. & Dalz is one of the many medicinal plants used for ages in folklore as male fertility enhancers. The current study evaluates the effect of the plant leaf extract on alcohol - induced reproductive toxicity in male rats.

**Methods:**

Thirty rats were randomly divided into six groups of five animals each; Group 1 (positive control) received normal saline only; Group 2 (ethanol alone) were given only 30 % ethanol orally at 7 ml/kg body weight per day, thrice in a week; Group 3, 4, 5 were given ethanol and co-treated with 50 mg/kg, 500 mg/kg and 1000 mg/kg body weight of leaf extract respectively while Group 6 were given ethanol and co-treated with a fertility drug, clomiphene citrate. All the drugs were given daily and the experiment lasted for twenty one consecutive days.

**Results:**

Alcohol ingestion resulted in a significant (*p* < 0.05) decrease in water, food intake and marked elevation of lipid peroxidation as assessed by the accumulation of malondialdehyde (MDA) in the reproductive tissues. Precisely, MDA level was elevated in the testis, epididymis, seminal vesicle and prostate gland by 81 %, 63 %, 95 % and 91 %, respectively. Furthermore, levels of total protein, reduced glutathione (GSH), vitamin C and activities of antioxidant enzymes in the reproductive tissues were significantly (*p* < 0.0001) reduced in ethanol-ingested rats. Interestingly, co-administration of *T. conophorum* with ethanol led to almost complete inhibition of lipid peroxidation thereby enhancing antioxidant status of the reproductive tissues.

**Conclusion:**

Overall, *T. conophorum* ameliorates oxidative reproductive toxicity induced by ethanol in male rats and its ameliorative effect comparable well with the fertility drug, clomiphene citrate.

## Background

Male infertility is a multifactorial disease, with numerous factors contributing to both reduced spermatogenesis and the production of dysfunctional sperm. Male infertility can be caused by various conditions such as genetic abnormality, infection of reproductive organ as well as oxidative stress due to a surplus of free radicals [1]. The free radicals inhibit the mitochondrial function of the sperm cells, retarding their movement and initiating teratozoospermia. This may explain the possible aetiologies for increasing cases of infertility among males [2].

The testicular free radical damage is due to higher level of polyunsaturated fatty acid (PUFA) which normally provides the sperm cells with the structural fluidity required to engage in the membrane fusion event (capacitation) associated with fertilization [3], low oxygen tension and lack of antioxidant defence mechanism [4], which renders it vulnerable to oxidative damage by chain reactions, a condition called lipid peroxidation [5]. Thus, the lipid layer was damaged and the efficiency of reproductive organ is lowered. Although sperm cells possess cytoplasmic antioxidant enzymes such as glutathione and superoxide dismutase, the amount is relatively small, thereby limiting the degree of protection conferred on the spermatozoa [6].

*Tetracarpidium conophorum* (Mull.Arg) Hutch & Dalziel (Euphorbiaceae) is one of those fertility enhancing plants used by traditional healers to treat infertility in males. It is locally cultivated mainly for the nuts which are cooked and consumed as snacks [7]. It is locally used by the elderly people for the treatment of constipation. The amino and fatty acids components of the nut are used for the treatment of prolonged and constant hiccups [8]. The barks are used in coffee as laxative and also chewed to reduce toothache. The leaves, bark and fruit of the plant are used medicinally and their uses include giddiness, toothache, eczema, pruritus, psoriasis, common cold and prostate cancer [9]. Also in West Africa, the leaves are used as male fertility agent and in the treatment of dysentery. Ikpemel *et al.* [10] reported the dietary effect of the plant seed on sperm quality and hormone profile in experimental animals. In our previous studies, we reported the presence of gallic acid, catechin, chlorogenic acid, caffeic acid, coumarin, rutin, quercitrin, quercetin, kaempferol and luteolin as the major constituents of the aqueous extract of the plant leaves [11] and we also established that *Tetracarpidium conophorum* elicited anti-peroxidative effect in reproductive tissues of Wistar rats [12], therefore in continuation with our search we tried to check the influence of the plant leaf extract on alcohol-induced oxidative reproductive toxicity, of which alcohol has been established by many authors to induce testicular toxicity [13–16].

## Methods

### Preparation of plant extract

Fresh samples of *T. conophorum* leaf were obtained from a farm land near Akure metropolis, Nigeria. The leaves were collected in May, 2014 and authentication of the sample was carried out at the Department of Plant Science, Ekiti State University by Mr Ajayi where voucher specimen (number UHAE 335) was deposited in the herbarium of the same Department.

The leaves were air dried, after which the dried samples were homogenized and kept dry in an air-tight container prior to the extraction. The plant material (50 g) was soaked in cold distilled water (1 l) for 24 h. The mixture was then filtered through Whatman No. 1 filter paper and the filtrate centrifuged at 805 × g for 10 min. The clear supernatant collected was freeze dried and labelled aqueous extract and stored in small, capped plastic container at 4 °C until required. The plant yield was 12.5 g dry powder/50 g powdered leaf. This was later reconstituted in water for subsequent analysis. The aqueous extract was prepared by dissolving the freeze dried extract sample in distilled water to yield concentration of 0.1 g/ml (100 mg/ml) as the stock concentration.

### Chemicals

Epinephrine, GSH, 5,5-dithio-bis-2-nitrobenzoic acid, hydrogen peroxide, NADP, NADPH, BSA, dithiothreitol, glutathione reductase, trichloroacetic acid (TCA) dinitrophenylhydrazine, thiourea, and thiobarbituric acid (TBA) were purchased from Sigma (St Louis, MO, USA). All other reagents were of analytical grade and were obtained from the Total Laboratory Technology (Gonubie, South Africa). The standard drug (Clomiphene citrate), fertility drug was gotten from CIPRA-MEDPRO (PTY) LTD, Rosen Heights, Pasita Street, Rosen Park, Bellville, 7530, Johannesburg, South Africa. Thirty percent (30 %) ethanol prepared from absolute ethanol (99.86 % v/v) with substance identification number 1170 manufactured by James Burrough (F.A.D. Ltd. UK) was used for the study.

### Experimental animals

All animal procedures have been approved and prior permission from the University of Fort Hare Animal Ethical Committee was obtained as per the prescribed guidelines. The bioethical allowance reference number was AFO021SAKO01. Thirty male albino Wistar rats (8–10 weeks old) weighing between 234–327 g were purchased from South African Vaccine Producers (Johannesburg, South Africa) and were housed at the University of Fort Hare Central Animal Unit. The rats were allowed to adapt to the new environment for at least two weeks before the experiment. They were kept under standard condition (inverted 12 h light/dark cycle), constant temperature (22 °C ± 2 °C) and humidity (70 % ± 4 %) with excess feeding of water and standard diet (Avi Products (Pty) Ltd. No. 21825) *ad libitum*. The animals were handled according to the guidelines of the National Research Council Guide for the Care and Use of Laboratory Animals [17]. Ethical care and handling of experimental animals was observed at all times. All the rats were provided with commercially available rat normal pellet diet (NPD) and water *ad libitum*. After two weeks of acclimatization the rats were then divided into six groups (1, 2, 3, 4, 5 and 6) using completely randomised design with five rats in each group. Group 1 (positive control) received normal saline only; Group 2 (ethanol alone) were given only ethanol; Group 3 (ethanol + 50 mg/kg BW of extract) were given ethanol and co-treated with 50 mg/kg body weight of leaf extract; Group 4 (ethanol + 500 mg/kg BW of extract) were given ethanol and co-treated with 500 mg/kg body weight of leaf extract; Group 5 (ethanol + 1000 mg/kg BW of extract) were given ethanol and co-treated with 1000 mg/kg of leaf extract while Group 6 (ethanol + 1.04 mg/kg BW of standard drug) were given ethanol and co-treated with a fertility drug, clomiphene citrate and the experiment lasted for twenty one consecutive days. The ethanol and ethanol treated groups (rats) were given ethanol solution according to the method of Dosumu *et al.* [18] with a slight modification. Briefly, 30 % ethanol was administered orally at 7 ml/kg body weight per day, thrice in a week [18] using a metal oropharyngeal cannula. Body weights were registered on every alternate day until the end of the experiment. Total quantity of food (g) and water consumed (ml) over the 24 h period was measured in each group daily. Extract and standard drug treatments were given daily by gavage using a metal oropharyngeal cannula as described in the protocols used by Akinola *et al.* [19]. After 21 days of the treatment period, the animals were anesthetized by diethyl ether and the tissue samples from reproductive tissues and accessory glands were collected for biochemical analysis.

### Necropsy

The animals were fasted overnight, weighed and sacrificed by decapitation 24 h after the last treatment and blood was collected by cardiac puncture. Testes, epididymes, seminal vesicles and prostate glands were removed and cleared of adhering tissues, washed in ice-cold 1.15 % potassium chloride and dried with blotting paper. The weights of the organs were recorded in gram (g) and also expressed as well as g/100 g body weight.

### Enzyme assay

The testes, epididymes, seminal vesicles and prostate glands were homogenized separately in 50 mM Tris–HCl buffer (pH 7.4) containing 1.15 % KCl and the homogenates were centrifuged at 10,000 g for 15 min at 4 °C. The supernatants were collected for the estimation of catalase (CAT) activity using hydrogen peroxide as substrate according to the method of Clairborne [20]. Superoxide dismutase (SOD) activity was determined by measuring the inhibition of autoxidation of epinephrine at pH 10.2 at 30 °C according to Misra [21]. Glutathione-S-transferase (GST) activity was estimated by the method of Habig *et al.* [22] using 1-chloro-2,4-dinitrobenzene (CDNB) as substrate. GPx was assayed by measuring the disappearance of NADPH at 35 °C according to Paglia & Valentine [23] and the unit is expressed as moles of NADPH oxidized per milligram of protein. Protein concentration was determined by the method of Lowry *et al.* [24].

### GSH assay

Reduced GSH was determined at 412 nm using the method described by Jollow *et al.* [25].

### Vitamin C content determination

Vitamin C content was determined at 520 nm using the method of Benderitter *et al.* [26].

### Lipid peroxidation assay

Lipid peroxidation was quantified as malondialdehyde (MDA) according to the method described by Farombi *et al.* [27] and expressed as µmol MDA/mg Protein.

### Statistical analysis

The data reported herein are the means of five replicates (*n* = 5). Means separation was done using Fisher’s protected least significant difference (LSD) test at (*p* < 0.05), (*p* < 0.01), (*p* < 0.001) and (*p* < 0.0001). One way analysis of variance was done to evaluate relationship between control and treated groups. All statistical analysis were done using JMP Release 10.0 statistical package (SAS Institute, Inc., Cary, North Carolina, USA, 2010).

## Results and discussion

Ingestion of rats with chronic ethanol for 21 days, as tested by us, induced a significant increase (14.44 %) in the body weight of the rat when compared with the control group (Table 1). This result is contrary to the report of Reidelberger *et al.* [28] who reported a weight loss in rats fed with alcohol. The weight gained by the rats may be due to the fact that alcohol act as a toxin and it has a priority treatment and goes straight to the liver to be metabolised ahead of the stored energy in the fat cell system thereby reducing (slow down) the resulted body metabolites plus the fact that the fat carry a lot of sugar which is also stored in fat cells system. It may be that the alcohol does two things, slows down the body ability to metabolize the fat or it makes the body to have more stored energy which is also fat. However, the co-treatment of rats with the *T. conophorum* extract for 21 days caused a significant decrease in their body weights (1.71, 6.85 and 0.34 %) at 50, 500 and 1000 mg/kg respectively while the standard drug caused a weight loss of 8.72 % when compared with the alcohol groups (14.44 %). Alcohol ingestion caused a decrease in the testis and other reproductive accessories (Table 1). The decrease in the weights of right testis, left testis, epididymis, seminal vesicle and prostate gland was almost 39 %, 54 %, 58 %, 73 % and 66 %, respectively. However, the drug and extract treatments showed noteworthy recovery in all the organs towards that of control groups whereas the extract at the highest concentration used in this study showed a more pronounced recovery effect in the weights of right testis, left testis, epididymis and prostate gland.

**Table 1:** Body and reproductive tissue weights of control and alcohol-induced male albino rats treated with the *T. conophorum* leaf extract

Parameters	Control	Alcohol group	50 mg/kg extract	500 mg/kg extract	1000 mg/kg extract	1.04 mg/kg drug
Initial body weight (g)	348 ± 8.66	284 ± 13.11	292 ± 21.03	292 ± 22.23	294 ± 18.52	298 ± 16.52
Final body weight (g)	368 ± 7.81	325 ± 9.07	297 ± 17.95	312 ± 16.52	295 ± 15.59	272 ± 18.94
Weight gain/loss (%)	5.75	14.44*	1.71*	6.85*	0.34*	−8.72*
Right testis						
Absolute weight (g)	2.03 ± 0.15	1.23 ± 0.03	1.73 ± 0.13	2.07 ± 0.12	2.08 ± 0.02	1.80 ± 0.13
Weight (g/ 100 g BW)	0.74 ± 0.00	0.32 ± 0.05*	0.49 ± 0.01*	0.56 ± 0.08	0.70 ± 0.03	0.72 ± 0.09
Left testis						
Absolute weight (g)	2.17 ± 0.45	0.99 ± 0.04*	1.67 ± 0.06*	2.14 ± 0.19	2.15 ± 0.08	2.07 ± 0.16
Weight (g/ 100 g BW)	0.69 ± 0.02	0.22 ± 0.02*	0.55 ± 0.06	0.57 ± 0.12	0.58 ± 0.02	0.51 ± 0.02
Epididymis						
Absolute weight (g)	1.47 ± 0.41	0.61 ± 0.05*	1.05 ± 0.16	1.40 ± 0.39	1.43 ± 0.08	1.42 ± 0.11
Weight (g/ 100 g BW)	0.50 ± 0.03	0.18 ± 0.05*	0.34 ± 0.02	0.37 ± 0.12	0.39 ± 0.05	0.31 ± 0.02
Seminal vesicle						
Absolute weight (g)	1.09 ± 0.41	0.29 ± 0.05*	0.40 ± 0.06*	0.95 ± 0.11	0.96 ± 0.08	0.96 ± 0.07
Weight (g/ 100 g BW)	0.46 ± 0.03	0.08 ± 0.01*	0.14 ± 0.02*	0.17 ± 0.03*	0.35 ± 0.04	0.15 ± 0.02*
Prostate gland						
Absolute weight (g)	1.20 ± 0.22	0.40 ± 0.07*	0.84 ± 0.18	1.16 ± 0.10	1.21 ± 0.18	1.17 ± 0.19
Weight (g/ 100 g BW)	0.60 ± 0.00	0.12 ± 0.05*	0.20 ± 0.03*	0.29 ± 0.03*	0.51 ± 0.06	0.26 ± 0.02*

Data are expressed as mean ± SD, *n* = 5. *Significant (*p* < 0.0001) compared with the control group

**Fig. 1:**
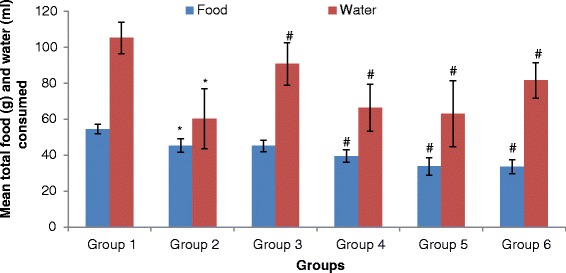
The mean total food and water intake of control and alcohol-induced male albino rats treated with the *T. conophorum* leaf extract. 1: Normal control, 2: Alcohol group, 3: Alcohol + 50 mg/kg of extract, 4: Alcohol + 500 mg/kg of extract, 5: Alcohol + 1000 mg/kg of extract, 6: Alcohol + 1.04 mg/kg of standard drug. Data are expressed as mean ± SD, *n* = 5. *Significantly different (*p* < 0.05) from control, ^#^Significantly different (*p* < 0.05) from alcohol group

We also compared the drinking and feeding patterns of control, alcohol, and alcohol co-treated with the extract and fertility drug groups and we discovered that alcohol ingested rats had low appetite for food and water as observed in the significantly lower (*p* < 0.05) water and food intake when compared with control group (Fig. 1). Our result was in agreement with the report of Caufield, [29]. Also, Richardson and Rumsey [30] found that fluid and food intake was reduced in rats when fed with ethanol as compared with when given ordinary drinking water even though the frequency was not affected. However, on co-treating the animals with the standard drug and extract, the water intake increased gradually towards the control group while the animals displayed more poor eating habit at 1.04 mg/kg of standard drug, 500 and 1000 mg/kg of extract as observed in the significantly lower food intake when compared with the alcohol group (Fig. 1).

**Fig. 2:**
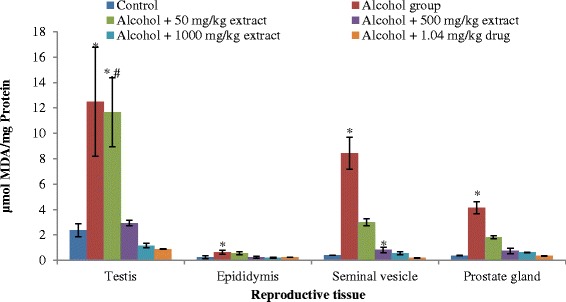
The MDA concentration in the reproductive tissues of control and alcohol-induced male albino rats treated with the *T. conophorum* leaf extract. Data are expressed as mean ± SD, *n* = 5. *Highly significant (*p* < 0.0001) compared with the control group. ^#^Significantly different (*p* < 0.05) from alcohol group

Ethanol-ingestion caused marked elevation of lipid peroxidation as assessed by the accumulation of malondialdehyde (MDA) in the testis and other accessory tissues of the rats (Fig. 2). Precisely, MDA level was elevated in the testis, epididymis, seminal vesicle and prostate gland by 81 %, 63 %, 95 % and 91 % respectively in alcohol-ingested rats relative to controls. Increased MDA levels in all the locations were markedly decreased by the co-administration of both standard drug and extract to the alcohol-induced rats. This increase was prevented on co-administration with the extract and standard drug at 50, 500, 1000 mg/kg of extrat and 1.04 mg/kg of drug by 6 %, 76 %, 91 %, 93 %; 9 %, 59 %, 67 %, 61 %; 64 %, 90 %, 93 %, 97 % and by 55 %, 81 %, 84 %, 91 % in the testis, epididymis, seminal vesicle and prostate gland respectively (Table 2). Alcohol has been established by many authors to induce lipid peroxidation in the reproductive tissue of rats [31–34].

**Table 2:** Percentage decrease in the level of MDA produced in the reproductive tissues of alcohol-induced male albino rats treated with the *T. conophorum* leaf extract

Treatment groups	Testis	Epididymis	Seminal vesicle	Prostate gland
Control (MDA level)^a^	3.21 ± 0.27	0.21 ± 0.00	0.64 ± 0.08	0.93 ± 0.12
Alcohol group (MDA level)^a^	12.48 ± 4.29	0.63 ± 0.15	8.42 ± 1.25	4.15 ± 0.46
Alcohol + 50 mg/kg extract	6.63	9.58	64.53	55.58
Alcohol + 500 mg/kg extract	76.45	59.42	90.24	81.86
Alcohol + 1000 mg/kg extract	90.70	67.48	93.33	84.89
Alcohol + 1.04 mg/kg drug	92.94	61.03	97.66	91.70

^a^μmol MDA/ mg protein

The result presented in Table 3 shows a highly significant (*p* < 0.0001) decrease in the levels of total protein, reduced glutathione (GSH) and vitamin C contents in the reproductive tissues of ethanol-ingested rats relative to controls. However, the co-treatment with the drug and extract restored the total protein, reduced glutathione and vitamin C contents in the reproductive tissues towards normalcy, but is still significantly lower than the control groups. There were no significant differences in the vitamin C content of the drug and extract co-treated rats relative to controls in all the reproductive tissues tested (Table 3).

**Table 3:** Total protein (TP), reduced glutathione (GSH) and vitamin C contents in the reproductive tissues of control and alcohol-induced male albino rats treated with the *T. conophorum* leaf extract

Parameters	Normal control	Ethanol group	Ethanol + 50 mg/kg extract	Ethanol + 500 mg/kg extract	Ethanol + 1000 mg/kg extract	Ethanol + 1.04 mg/kg drug
Testis						
TP^a^	15.00 ± 0.00	4.25 ± 0.02*	4.66 ± 0.08	6.33 ± 0.90**	11.66 ± 0.72**	13.75 ± 1.25**
GSH^b^	23.90 ± 1.00	3.76 ± 0.46*	7.33 ± 0.20**	8.12 ± 0.21**	9.95 ± 0.03**	14.52 ± 0.21**
Vitamin C^c^	3.10 ± 1.80	0.33 ± 0.04*	0.68 ± 0.00	0.82 ± 0.29	1.12 ± 0.16**	1.04 ± 0.23**
Epididymis						
TP^a^	120.00 ± 10.00	32.91 ± 2.25*	34.16 ± 1.44	83.75 ± 1.25**	110.41 ± 0.72**	101.66 ± 22.02**
GSH^b^	10.42 ± 0.00	3.27 ± 0.00*	3.50 ± 0.04	3.79 ± 0.02	9.26 ± 0.11**	4.56 ± 0.01
Vitamin C^c^	2.44 ± 0.00	0.91 ± 0.09	1.36 ± 0.05	1.54 ± 0.02	2.05 ± 0.21	2.08 ± 0.01
Seminal vesicle						
TP^a^	58.33 ± 13.45	8.66 ± 0.72*	8.58 ± 2.60	10.83 ± 0.72	25.83 ± 2.60**	37.08 ± 6.63**
GSH^b^	6.21 ± 0.22	2.52 ± 2.04*	3.42 ± 0.40	4.83 ± 0.07	5.28 ± 0.12**	5.56 ± 0.01**
Vitamin C^c^	1.57 ± 0.06	0.68 ± 0.06	0.83 ± 0.13	0.94 ± 0.02	1.29 ± 0.01	1.30 ± 0.01
Prostate gland						
TP^a^	61.25 ± 1.25	8.16 ± 0.72*	10.25 ± 2.50	18.75 ± 1.25**	26.25 ± 3.92**	31.66 ± 4.72**
GSH^b^	10.11 ± 0.04	4.55 ± 0.31*	5.57 ± 0.03	6.46 ± 0.03	8.50 ± 1.01**	8.78 ± 0.04**
Vitamin C^c^	1.52 ± 0.73	0.38 ± 0.03	0.49 ± 0.02	0.59 ± 0.19	0.77 ± 0.03	0.85 ± 0.00

^a^(mg/ml), ^b^(μg/ mg protein), ^c^(mmol/ mg protein). Data are expressed as mean ± SD, *n* = 5. *Highly Significant (*p* < 0.0001) compared with the control group, **Significant (*p* < 0.05) compared with the alcohol group

Free radical scavenging enzymes such as SOD, CAT and glutathione peroxidase (GPx) are the first line of defence against oxidative injury. SOD protects tissues against oxygen free radicals by catalysing the removal of superoxide radical, converting it into H_2_O_2_ and molecular oxygen, which both damage the cell membrane and other biological structures [35]. These radicals (H_2_O_2_ and lipid peroxide) are readily degraded by catalase and glutathione peroxidase using reduced GSH to non-toxic alcohol. Catalase is a haem-protein, which is responsible for the detoxification of significant amounts of H_2_O_2_ [36].

**Fig. 3:**
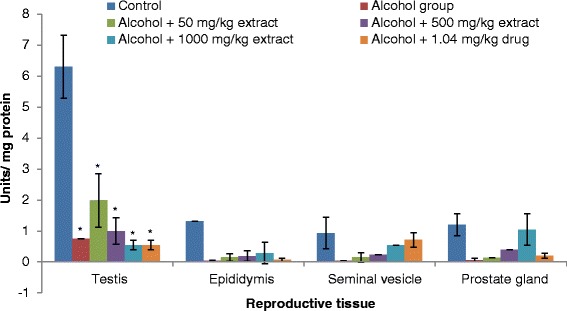
Superoxide dismutase (SOD) levels in the reproductive tissues of control and alcohol-induced male albino rats treated with the *T. conophorum* leaf extract. Data are expressed as mean ± SD, *n* = 5. *Highly significant (*p* < 0.0001) compared with the control group

**Fig. 4:**
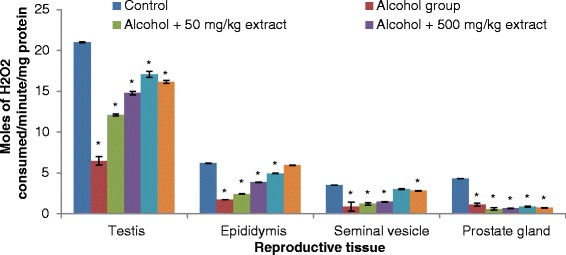
Catalase (CAT) levels in the reproductive tissues of control and alcohol-induced male albino rats treated with the *T. conophorum* leaf extract. Data are expressed as mean ± SD, *n* = 5. *Highly significant (*p* < 0.0001) compared with the control group

**Fig. 5:**
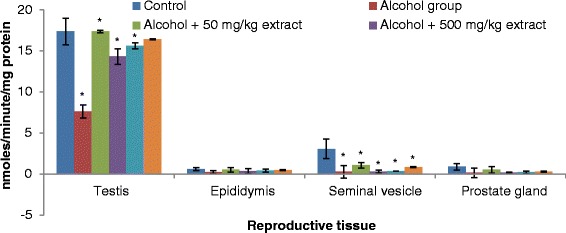
Glutathione-S-transferase (GST) levels in the reproductive tissues of control and alcohol-induced male albino rats treated with the *T. conophorum* leaf extract. Data are expressed as mean ± SD, *n* = 5. *Highly significant (*p* < 0.0001) compared with the control group

**Fig. 6:**
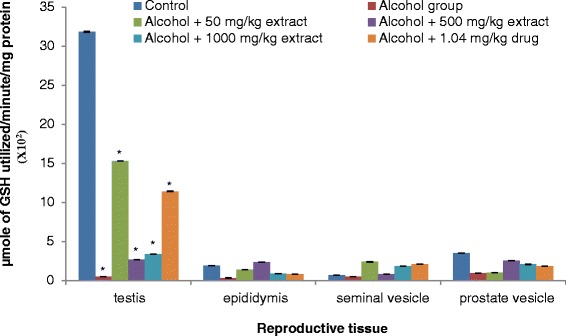
Glutathione peroxidase (GPx) levels in the reproductive tissues of control and alcohol-induced male albino rats treated with the *T. conophorum* leaf extract. Data are expressed as mean ± SD, *n* = 5. *Highly significant (*p* < 0.0001) compared with the control group

Our results revealed that testis, epididymis, seminal vesicle and prostate gland antioxidant enzymes (SOD, catalase, GST and GPx) activities significantly decreased in rats ingested with ethanol compared to normal controls (Figs. 3, 4, 5 and 6). The decrease in the activities of these enzymes could be attributed to the excessive utilization of these enzymes in inactivating the free radicals generated from ethanol ingestion [37] or insufficient availability of GSH. This observation is further substantiated by the elevated malondialdehyde levels (Fig. 2). Our results are in agreement with those of others, who studied and established the effect of ethanol on testis antioxidant enzymes system [14, 31, 38, 39] and this was confirmed in this study, thus chronic use of alcohol may lead to reproductive failure. Co-administration of ethanol-ingested rats with extracts of leaf of *T. conophorum* significantly restored the reduction in the level of SOD, catalase, GST and GPx in the testis, epididymis, seminal vesicle and prostate gland of rats in dose dependent manner to value that was statistically similar to controls. The protective effect of the extract is comparable with the fertility drug, clomiphene citrate. Our results indicate that *T. conophorum* had a free radical scavenging activity which probably provides reproductive tissues protection from chronic alcohol-intoxication.

**Fig. 7:**
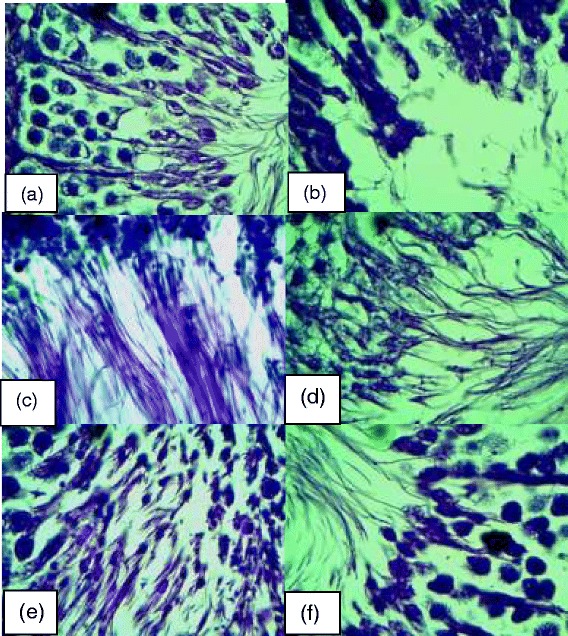
Representative photomicrographs of the testis sections (viewed under X100 magnification) from control, alcohol, alcohol plus extract and alcohol plus standard drug. (**a**) control rat showing normal lumen with plenty of intact sperm cells (**b**) alcohol-induced rat showing degenerated lumen with loss of sperm cells (**c**) 50 mg/kg extract treated alcohol-induced rat showing improvement in the degenerated lumen with little traces of sperm cells (**d**) 500 mg/kg extract treated alcohol-induced rat showing nearly normal lumen filled with sperm cells (**e**) 1000 mg/kg extract treated alcohol-induced rat showing normal lumen filled with sperm cells (**f**) drug treated alcohol-induced rat showing normal lumen filled with sperm cells

The histopathological slides viewed under X100 magnification revealed the lumen of the seminiferous tubules of both control, alcohol, and alcohol co-treated with the extract and fertility drug groups (Fig. 7). The photomicrographs of the testis sections of alcohol-ingested rats revealed degenerated lumen with loss of sperm cells. These observations were significantly attenuated in slides obtained from alcohol co-treated with the fertility drug and extract rats in a concentration dependent manner. In the present study, we found that co-administration of *T. conophorum* to ethanol-ingested rats significantly decreased the lipid peroxidation and caused marked elevation of total protein, vitamin C, GSH, SOD, catalase, GST and GPx in the reproductive tissues. Furthermore, its co-administration improved the degenerated lumen of the testis.

## Conclusion

In conclusion, the observed ameliorative effect of *T. conophorum* in this study may be due to its antioxidant properties which may be involved in the scavenging of radical species generated by ethanol which in turn may be linked to its phytochemical constituents. From these findings, it can be inferred that *T. conophorum* positively modulates the antioxidant status and regenerates the reproductive tissues of ethanol-ingested rats to near normal.
